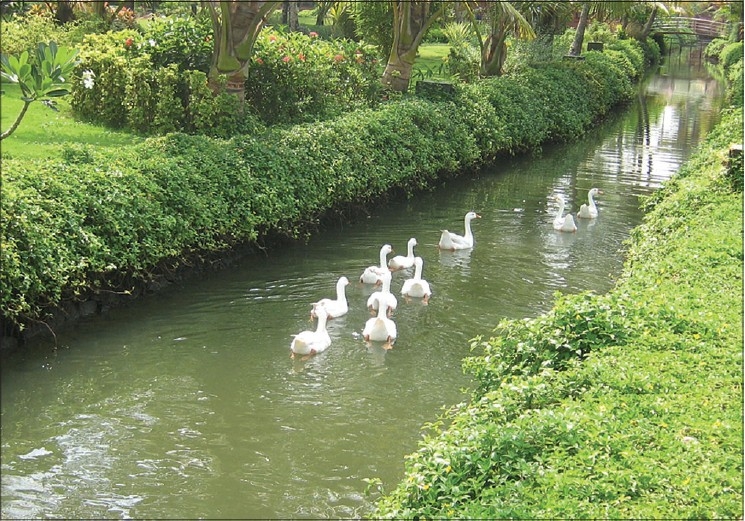# No footprints, yet after the leader

**Published:** 2009

**Authors:** Jacob George

**Affiliations:** Department of Neurology, Medical College, Kottayam, Kerala, India. E-mail: drjacobgeorge35@yahoo.co.in